# The Needs and Resources of Drug Information at Community Pharmacies in Gondar Town, Northwest Ethiopia

**DOI:** 10.1155/2017/8310636

**Published:** 2017-08-29

**Authors:** Dessalegn Asmelashe Gelayee, Gashaw Binega Mekonnen, Mequanent Kassa Birarra

**Affiliations:** ^1^Department of Pharmacology, College of Medicine and Health Sciences, University of Gondar, Gondar, Ethiopia; ^2^Department of Clinical Pharmacy, College of Medicine and Health Sciences, University of Gondar, Gondar, Ethiopia

## Abstract

**Background:**

Community pharmacists are in a key position to provide information on drugs and thus promote the rational use of drugs.

**Objectives:**

The present study was designed to determine the needs and resources of drug information in community pharmacies.

**Methods:**

A prospective institution based cross-sectional study was carried out and data were collected on 48 community pharmacists in Gondar, Northwest Ethiopia, using interviewer administered structured questionnaire.

**Results:**

Almost all pharmacists (*N* = 47, 97.9%) often receive drug related queries and these were mainly from consumers (*N* = 41, 85.4%). While most questions relate to drug price (*N* = 29, 60.4%) and dosage (*N* = 21, 43.8%), the information resources mainly referred to were drug package inserts and national standard treatment guidelines. However, limited availability of information resources as well as limited ability to retrieve relevant information influenced the practice of pharmacists. Female pharmacists claimed better use of different information resources than males (*P* < 0.05).

**Conclusions:**

Community pharmacists in Gondar, Northwest Ethiopia, are often accessed for drug related information. But there are limitations in using up to date and most reliable resources. Therefore, intervention aimed at improving pharmacists' access to and evaluation of drug information is urgently needed.

## 1. Introduction

Pharmacy profession is very dynamic and pharmacists' role is improving with the expansion of the scope of services. It has evolved from that of a compounder and supplier of pharmaceutical products towards that of a provider of services and information and ultimately that of a provider of patient care [1]. Appropriate drug information is vital for the correct use of drugs and improves patient outcome. The International Pharmaceutical Federation (FIP) states that it is the responsibility of the pharmacist to ensure that the patient receives the required information for the quality use of medications [2]. On the other hand, failure to provide quality drug related information can have several harmful outcomes. It is estimated that between one-third and half of medicines used all over the world are wasted, with impacts on economy and health [3]. A study done in Ethiopia reported that lack of drug information related to the adverse effects among the laypeople contributed to high involvement in self-medication practice [4]. Common situations such as adverse drug reactions, drug-drug interactions, and drug use during pregnancy require the access of drug information sources which are up-to-date, accurate, user-friendly, and trustworthy [5].

According to the Food, Medicine and Healthcare Administration and Control Authority (FMHACA) of Ethiopia, persons involved in drug dispensing have to make themselves up to date with drug information in order to provide this information for patients, other health care professionals, and the general public [6]. A study done in Ethiopia reported that 76% of 50 prescribers surveyed reported having access to up-to-date drug information, while from 30 dispensers, 43.3% did not get access to up-to-date drug information [7]. Worldwide, community pharmacists account for 55% of pharmacists and are one of the most accessible healthcare practitioners [8]. They are in an ideal position to provide drug related information to the general public and health care professionals. However, lack of ability to correctly address drug related questions may raise questions from patients, practitioners, and regulators. It may also lead to doubts about the pharmacist's professionalism. Therefore, the main purpose of the present study is to determine the needs and resources of drug information at community pharmacies in Gondar Town, Northwest Ethiopia.

## 2. Methods

Institution based cross-sectional study was conducted among pharmacists working in the community pharmacies in Gondar Town, Northwest Ethiopia, in January 2017. About 53 medication retail outlets (19 pharmacies and 34 drug stores) were registered in the town in 2014. Community pharmacist in the present study refers to at least diploma holders in pharmacy education and community pharmacy refers to both drug stores and pharmacies. Data collected using a structured interviewer-administered questionnaire was analyzed with statistical package for social sciences (SPSS) version 20.0 for windows (SPSS Inc., Chicago, IL). The data collection instrument (attached as supplementary material, in Supplementary Material available online at https://doi.org/10.1155/2017/8310636) was developed based on previous studies [9–11]. It comprises closed types of questions with yes/no and 3-point Likert scale responses (never, sometimes, and always): Part I: sociodemographic characteristics; Part II: type of drug related questions received; Part III: type of drug information resources used; and Part IV: barriers limiting pharmacists' ability to fulfill drug information needs at the practice site. The questionnaire was evaluated and approved for face validity by three senior pharmacists who are researchers and academicians. Then it was pretested on 5 pharmacy technicians working as part time in private pharmacies and modifications were made before data collection. About 48 pharmacists working in community pharmacies in the town gave their consent to be included in the study. The reliability assessment of the different subcomponents of the questionnaire after data collection revealed a Cronbach's alpha value of 0.865 for type of drug related questions received (10 items), 0.792 for type of drug information resource used (10 items), and 0.784 for barriers limiting pharmacists' ability to fulfill drug information needs at the practice site (5 items). The results were described in terms of frequencies, percentages, and means. The relationships among variables were analyzed by using Mann–Whitney *U* test and Pearson's Chi-square test of independence with a *P* value < 0.05 considered statistically significant. An ethical clearance was taken from the Ethical Review Committee of School of Pharmacy, University of Gondar, and all respondents gave their consent before participation in the study.

## 3. Results

### 3.1. Sociodemographic Characteristics

Forty-eight community pharmacists participated in the study and the majority were male (*N* = 31, 64.6%), were 23–28 years old (*N* = 27, 56.3%), were at least B.pharm degree holders (*N* = 28, 58.4%), had work experience of 4 years and below (*N* = 25, 52.1%), and were employees (*N* = 30, 62.5%). Absence of Internet service in the pharmacy was reported by 41 (85.4%) respondents. Almost all (*N* = 47, 97.9%) pharmacists often receive drug related questions. Majority (*N* = 41, 85.4%) of them receive inquiries from consumers (Table 1).

As shown in Table 2, the types of drug related questions always presented to the pharmacists in descending order were related to price (*N* = 29, 60.4%), dosage (*N* = 21, 43.8%), and drug identification (*N* = 14, 29.2%), whereas inquiries related to poisoning and herbal drugs were less frequent and never received by 21 (43.8%) and 25 (52.1%) pharmacists.

**Figure 1:**
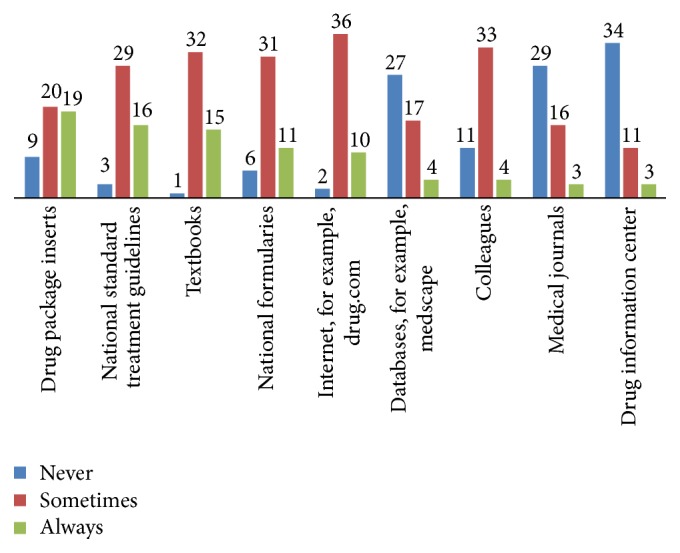
*Drug information resources used by the community pharmacists *(*N* = 48). The data presented refers to community pharmacists working in Gondar Town and was generated in January 2017 in Gondar, Ethiopia.

The types of information resources always used by the pharmacists, when put in descending order, were drug package inserts, national standard treatment guidelines, and pharmacology text books. Drug information center was never used by the majority (*N* = 34, 70.8%) of respondents, Figure 1.

All the pharmacists reported using drug information resources to refer to adverse drug reaction, drug identification, use, and dose. Twenty-seven (56.2%) respondents also check stability and compatibility issues.

**Figure 2:**
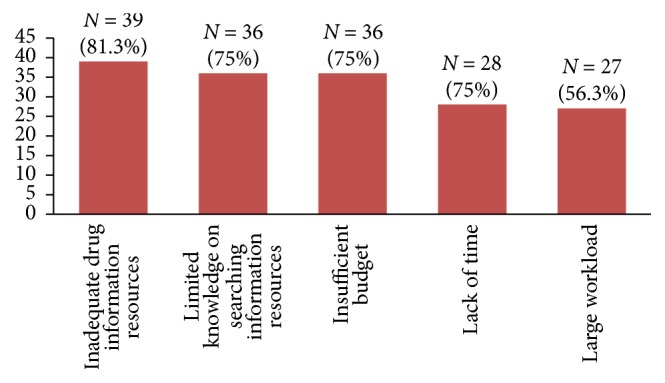
*Barriers limiting pharmacists' ability to fulfill drug information needs at the practice site *(*N* = 48). The data presented refers to community pharmacists working in Gondar Town and was generated in January 2017 in Gondar, Ethiopia.

The respondents identified several barriers that limit their ability to fulfill drug information needs at the practice site. The top three barriers were inadequate drug information resources (*N* = 39, 81.3%), limited knowledge on searching information resources (*N* = 36, 75.0%), and insufficient budget (*N* = 36, 75.0%) (Figure 2).

Based on the Mann–Whitney *U* test, group based differences were found for sex of respondents and pharmacy ownership status regarding the type of drug related questions received. Females reported frequent receipt of drug dosage (females *N* = 17, mean rank = 29.53; males *N* = 31, mean rank = 21.74; *U* = 178, *z* = −2.145, *P* = 0.032) and herbal drug related questions (females *N* = 17, mean rank = 30.26; males *N* = 31, mean rank = 21.34; *U* = 165.5, *z* = −2.419, *P* = 0.016) compared to the male pharmacists. The employees also receive herbal drug related questions more frequently than the owners (employees *N* = 30, mean rank = 27.48; owners *N* = 18, mean rank = 19.53; *U* = 180.5, *z* = −2.182, *P* = 0.029).

The Mann–Whitney *U* test also revealed group based differences for sex and additional work experience of respondents regarding the type of information resource used. Females reported more frequent use than males of text book (females *N* = 17, mean rank = 30.25; males *N* = 31, mean rank = 21.29; *U* = 164, *z* = −2.614, *P* = 0.009), Internet (females *N* = 17, mean rank = 28.62; males *N* = 31, mean rank = 22.24; *U* = 193.5, *z* = −2, *P* = 0.045), databases (females *N* = 17, mean rank = 29.47; males *N* = 31, mean rank = 21.77; *U* = 179, *z* = −2.066, *P* = 0.039), and drug information center (females *N* = 17, mean rank = 29.32; males *N* = 31, mean rank = 21.85; *U* = 181.5, *z* = −2.222, *P* = 0.026). Those with additional experience also more often used national treatment guidelines (*P* = 0.049) and drug package inserts (*P* = 0.035) than their counter groups.

Pearson's Chi-square test of independence was run to find out if sociodemographic factors are associated with the perception of respondents on barriers to achieving information need. Those lacking additional work experience perceived workload as a barrier than the counter groups (those with no additional experience *N* = 18 (69%), those with additional experience *N* = 9 (40.9%), *χ*^2^ = 3.884, df = 1, *P* = 0.049).

## 4. Discussion

Community pharmacists can provide information on medicines necessary to other health care professionals and to patients and use it in promoting the rational use of drugs [12]. Increased accessibility of community pharmacists is a good opportunity to achieve the above mission. A 2010 study in Ethiopia identified that community pharmacy accounted for 18.5% of the total 1898 pharmacists in the nation [13]. On the other hand, consumers' expectation is very high from community pharmacist including provision of drug related information. A study done in Gondar Town, Northwest Ethiopia, reported that 339 (80.7%) clients expect pharmacists to counsel/advise them in detail about their medications [14].

The findings of the present study show that community pharmacists in Gondar Town, Northwest Ethiopia, often receive drug related questions, most of which were directed from the customers and fellow pharmacists. Thus, it seems that these pharmacists are easily accessible by the clients [8] and there are strong intraprofessional communications among pharmacists more than with other health care professionals. Improving access to Internet service in the pharmacy setting may help pharmacists to remain updated on the science of drugs. Most common questions were related to drug price and dosage. Clients might be curious of drug prices in the community pharmacies since drugs constitute as high as 60%–90% of household health expenses in developing countries [15] and the drug prices are continuously on the rise. Drug price related information supplied to consumers and health professionals by pharmacists may enable patients receive the right drugs at reasonable cost, thereby improving rational drug use. Dosage related drug questions are more commonly encountered than those related to efficacy, adverse drug reaction, contra indication, and drug interaction. This strengthens the report by Ax et al. [16]. However, inquiries related to drug poisoning and herbal drugs were very uncommon and never received by 21 (43.8%) and 25 (52.1%) pharmacists. It needs to be researched why pharmacists are less consulted about herbal drugs. Pharmacists may be considered as less knowledgeable on herbal drugs. A study done in Qatar and Nigeria reported poor knowledge of pharmacists with regard to herbal drugs [17, 18]. In the present study, females more frequently received questions related to drug dosage (*P* = 0.032) and herbal drugs (*P* = 0.016) compared to male pharmacists. The employees also received herbal drug related questions more frequently than the owners (*P* = 0.029).

Types of information resources always used by the pharmacists, when put in descending order, were drug package inserts, national standard treatment guidelines, and text books. A study done in Nigeria reported that majority of pharmacists (*N* = 42; 72.8%) believe that information from drug package inserts was precise and used it as a reference [19]. A similar belief and easy availability might be attributed to the high use of drug package inserts as drug information resources in the present study. However, pharmacists need to be informed on the possibility of partiality of information obtained from pharmaceutical industries in the form of package inserts. Thus, using independent sources of information is crucial. Reference texts are also reported in previous studies as widely used sources [20, 21]. Despite the presence of drug information center in Gondar University hospital, the majority (*N* = 34, 70.8%) of respondents never used it for their drug information need. The reasons for underutilization may require further studies. A study done in Uganda in 2005 reported that out of 297 queries received in a pilot DIC operated by a pharmacist and a physician, only 1 (0.3%) was from drug shops and 26 (8.8%) were from pharmacists [22]. In the present study, females reported significantly more frequent use than males of textbook (*P* = 0.009), Internet (*P* = 0.045), databases (*P* = 0.039), and drug information center (*P* = 0.026). Those with additional experience also more often used national treatment guidelines (*P* = 0.049) and drug package inserts (*P* = 0.035) than their counter groups.

The respondents in the present study often use the different resources to address a variety of drug related information needs. All the pharmacists reported to use the resources to look for adverse drug reaction, drug identification, and therapeutic use and dose. This strengthens the findings of Hennigen et al. [21].

Several barriers that limit the pharmacists' ability to fulfill drug information needs at the practice site were identified. The most important barrier was limited presence of drug information resources and this strengthens findings from previous studies [21, 23, 24]. In addition, limitations on ability to search information resources and insufficient budget were identified as important barriers. Capacity building strategies such as continuing education and stimulating the use of drug information center may improve access to and utilization of drug information by community pharmacists.

## 5. Limitations of the Study

This study may not be generalized to the nation's community pharmacists as a whole because of small sample size.

## 6. Conclusions

The key finding of the present study is that pharmacists are more often accessed especially by consumers and fellow pharmacists for drug related questions mainly on drug pricing and dosage. Drug package inserts, treatment guidelines, and textbooks are the main information sources but there is less utilization of drug information center, drug data bases, and journals. The principal barriers identified were scarcity of such information resources as well as limited ability in accessing them. These findings justify the need to build capacity of community pharmacists to improve access and utilization of up-to-date drug information resources since it enhances their contribution in promoting rational drug use. The government and educators shall work more in this regard.

## Supplementary Material

This questionnaire is designed to evaluate the need and resources of drug information in community pharmacies of Ethiopia.

## Figures and Tables

**Table 1:** Sociodemographic characteristics of respondents (*N* = 48).

Variables	*N* (%)
Sex	
Female	17 (35.4)
Male	31 (64.6)
Age (years)	
29.7 ± 5.8 (mean ± SD)	
23–28 years	27 (56.3)
29–51 years	21 (43.8)
Educational level	
Diploma	20 (41.6)
B.pharm	27 (56.3)
M.S.	1 (2.1)
Work experience in community pharmacy (years)	
4.8 ± 3.2 (mean ± SD)	
1–4 years	25 (52.1)
5–16 years	22 (47.9)
Pharmacy ownership	
Owner	18 (37.5)
Employees	30 (62.5)
Additional work experience	
Yes	22 (45.8)
No	26 (54.2)
Internet service to the pharmacy	
Yes	7 (14.6)
No	41 (85.4)
Do you often receive drug related question	
Yes	47 (97.9)
No	1 (2.1)
From whom do you receive such questions	
Consumer	41 (85.4)
Pharmacists	18 (37.5)
Physicians	9 (18.8)
Nurses	5 (10.4)

The data presented refers to community pharmacists working in Gondar Town and was generated in January 2016 in Gondar, Ethiopia.

**Table 2:** Type of drug related questions received by the respondents (*N* = 48).

Type of drug related questions	Response *N* (%)		
	Never	Sometimes	Always
Price	5 (10.4)	14 (29.2)	29 (60.4)
Dosage	0	27 (56.2)	21 (43.8)
Drug identification	2 (4.2)	32 (66.7)	14 (29.1)
Efficacy of drugs	8 (16.6)	27 (56.3)	13 (27.1)
Adverse drug reactions	4 (8.3)	33 (68.8)	11 (22.9)
Contraindications	4 (8.4)	34 (70.8)	10 (20.8)
Drug interactions	7 (14.6)	34 (70.8)	7 (14.6)
Pharmaceutical compatibility and stability	32 (66.7)	11 (22.9)	5 (10.4)
Poisoning	21 (43.8)	26 (54.1)	1 (2.1)
Herbal drugs	25 (52.1)	22 (45.8)	1 (2.1)

*Note*. Three-point Likert scale (1 = never, 3 = always). The data presented refers to community pharmacists working in Gondar Town and was generated in January 2016 in Gondar, Ethiopia.
