# Supplementary material for: The Needs and Resources of Drug Information at Community Pharmacies in Gondar Town, Northwest Ethiopia
Source: Biomed Res Int. 2017 Aug 29;2017:8310636. doi: 10.1155/2017/8310636 (PMC5603327; doi:10.1155/2017/8310636)
Supplement: Supplementary file 1 — This questionnaire is designed to evaluate the need and resources of drug information in community pharmacies of Ethiopia. [file 8310636.f1.doc]

**The needs and resources of drug information at community pharmacies in Northwest Ethiopia**

**Part I. Socio-demographic characterstics**

1. **Sex:** female male
2. **Age (yr):**________
3. **Level of education:** diploma B.Pharm MSc
4. **Experience in community pharmacy (yr):**____________
5. **Employment status:** employee owner
6. **Additional work experience** yes no
7. **Internet service to the pharmacy** yes no
8. **Have you ever received drug related question?** yes no
9. **If your response to Qn 8 is yes, from whom you did you receive such questions?**

consumers pharmacists nurses physicians

Others_______________________________________________

**Part II. Type of drug related questions presented to community pharmacists**

| **Questions are related to** | **Response** | | |
| --- | --- | --- | --- |
|  | **Never** | **Sometimes** | **Always** |
| Dosage |  |  |  |
| Drug identification |  |  |  |
| Efficacy of drugs |  |  |  |
| Adverse drug reactions |  |  |  |
| Contraindications |  |  |  |
| Drug interactions |  |  |  |
| Pharmaceutical compatibility and stability |  |  |  |
| Price |  |  |  |
| Poisoning |  |  |  |
| Herbal drugs |  |  |  |

**Part III. Types of medicine information resources used by community pharmacists**

| **Information resources** | **Response** | | |
| --- | --- | --- | --- |
|  | **Never** | **Sometimes** | **Always** |
| Text books |  |  |  |
| National Standard treatment Guidelines |  |  |  |
| National Formularies |  |  |  |
| Internet |  |  |  |
| computer databases |  |  |  |
| Medical journals |  |  |  |
| Drug information center eg Gondar University Hospital |  |  |  |
| Colleagues |  |  |  |
| Pharmaceutical company representatives and company literature |  |  |  |
| Drug package inserts |  |  |  |

**Part IV: Barriers limiting pharmacists’ ability to fulfill drug information needs at the practice sites**

| **Barriers** | **Response** | |
| --- | --- | --- |
|  | **Yes** | **No** |
| Inadequate drug information resources |  |  |
| Insufficient budget |  |  |
| Lack of time |  |  |
| Large workload |  |  |
| Limited knowledge on searching information resources |  |  |
| Others (specify) |  |  |
